# G-Protein-Coupled Receptor Class C Group 6 Member A (GPRC6A) and Serine Protease Inhibitor, Kazal Type 1 (SPINK1) Genotypes Associated With Refractory Functional Dyspepsia: A Potential Link to Endosonographic Features

**DOI:** 10.7759/cureus.88222

**Published:** 2025-07-18

**Authors:** Sakura Higashida, Seiji Futagami, Ken Nakamura, Shuhei Agawa, Mayu Habiro, Rie Kawawa, Takeshi Onda, Tomohide Tanabe, Nobue Ueki, Masanori Atsukawa

**Affiliations:** 1 Division of Gastroenterology, Nippon Medical School, Tokyo, JPN

**Keywords:** early chronic pancreatitis, endoscopic ultrasonography, gprc6a, polymorphism, refractory functional dyspepsia, spink1

## Abstract

Background: This study aims to determine the associations between single-nucleotide polymorphisms (SNPs) in *G-protein-coupled receptor class C group 6 member A​​​​​​*​ (*GPRC6A)* and *serine protease inhibitor, Kazal type 1 *(*SPINK1)* genes and the clinical characteristics, including clinical symptoms, pancreatic enzyme abnormalities, and exocrine pancreatic function, with refractory functional dyspepsia (R-FD).

Methods: Around 97 patients with FD and 74 R-FD were recruited. Five pancreatic enzymes were measured. DNA was isolated from blood or duodenal tissues. Endoscopic ultrasonography (EUS) was performed using Olympus EUS (GF-UCT 260; Olympus, Tokyo, Japan) under conscious sedation in patients with R-FD. Exocrine or endocrine pancreatic function was estimated.

Results: Significant differences (p < 0.001) were observed in the ratio of abnormal pancreatic enzyme levels between patients with R-FD and those with FD. However, no significant differences in the distribution of *GPRC6A* and *SPINK1* genotypes were observed in the FD and R-FD groups. EUS score in the GG genotype was significantly higher than in the CC or CG genotypes (p = 0.036 and p = 0.031, respectively) in *GPRC6A* in R-FD. In addition, the lobularity in GG genotype in *GPRC6A* in R-FD was also significantly higher (p = 0.023 and p = 0.027, respectively) than that in CC or CG.

Conclusion: Significant differences (p < 0.001) were observed in the ratio of abnormal pancreatic enzyme levels between patients with R-FD and those with FD. *GPRC6A* genotype was significantly associated with EUS features, and further studies will be needed to clarify the significant association between* GPRC6A *genotype and EUS score.

## Introduction

Talley et al. defined functional dyspepsia (FD) as persistent epigastric pain or recurrent abdominal discomfort without evidence explaining these symptoms as an organic disease [1]. Stanghellini et al. also reported bothersome symptoms, including epigastric pain, epigastric burning, postprandial fullness, and early satiety, in patients with FD according to Rome IV criteria [1]. Several recent studies have investigated the treatment of patients with FD. Vonoprazan, proton pump inhibitors, and prokinetics are widely used to treat patients with FD. However, some patients with FD are resistant to these treatments. Refractory FD (R-FD) is defined as bothersome symptoms for at least six months despite at least two conventional medical treatments such as vonoprazan, proton pump inhibitors, mosapride, acotiamide, and *H. pylori* eradication [2]. Although many doctors have trouble with R-FD, data concerning the precise pathophysiology and useful treatment of R-FD are not available.

Patients with R-FD are diagnosed with celiac disease based on duodenal biopsies [3] and non-celiac gluten sensitivity [4]. We have reported that patients with vonoprazan- and acotiamide-R-FD frequently exhibit pancreatic enzyme abnormalities, such as a high ratio of trypsin abnormalities [5]. These trypsin abnormalities in the duodenum may react with protease-activated receptor 2 (PAR2) in the epithelium, leading to duodenal inflammation [6]. Considering the above reports, excessive release of pancreatic enzymes, such as trypsin, may be associated with the pathophysiology of patients with R-FD through pancreatic enzyme abnormality-related duodenal inflammation. In our study, we excluded early chronic pancreatitis from patients with R-FD with a score of < 2 on endoscopic ultrasonography (EUS) [7]. Since vonoprazan- and acotiamide-R-FD highly exhibited pancreatic enzyme abnormalities, we investigated the association between single-nucleotide polymorphisms (SNPs) associated with pancreatitis, such as *G-protein-coupled receptor class C group 6 member A​​​​​​​ (GPRC6A)* and *serine protease inhibitor, Kazal type 1 (SPINK1)* [8,9], and R-FD using EUS.

In this study, we aimed to investigate the associations between genotypes of *SPINK1* [9] and *GPRC6A* [8] and the pathophysiology of R-FD, including clinical symptoms, pancreatic enzyme abnormalities, exocrine pancreatic function, and endosonographic features.

## Materials and methods

Patients

**Figure 1:**
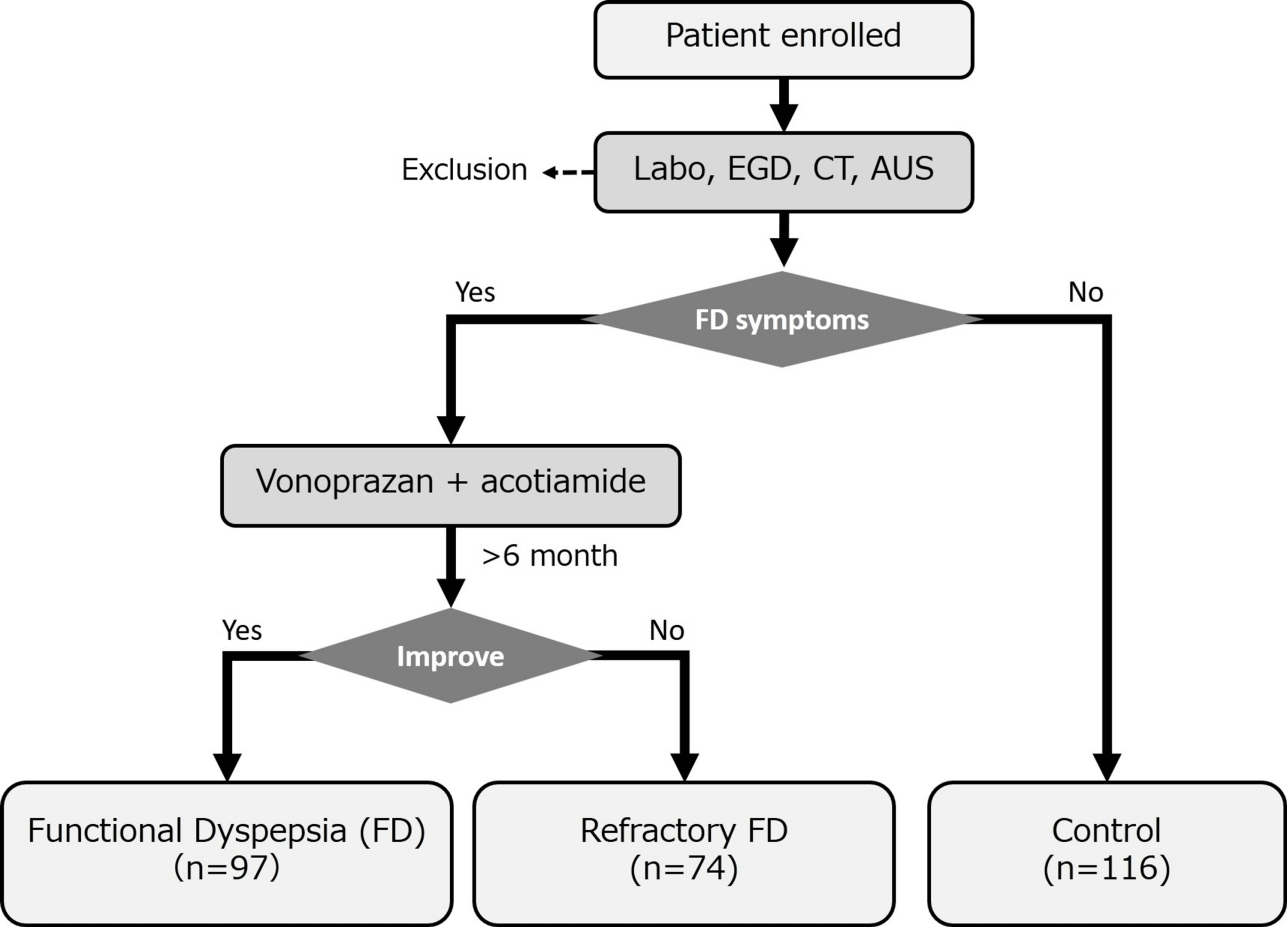
Study protocol AUS: abdominal ultrasound; EGD: esophagogastroduodenoscopy

In total, 287 patients were enrolled in this study: 74 in the R-FD group, 97 in the FD group, and 116 in the control group. Vonoprazan and acotiamide refractory patients were defined as the R-FD group (Figure 1) [2].

The control group was defined by no FD symptoms and no organic diseases examined by ultrasound (US), esophagogastroduodenoscopy (EGD), and CT. The Ethics Review Committee (approval no. M-2023-139) of the Nippon Medical School Hospital approved this study. Patients who participated in the study visited Nippon Medical School Musashi Kosugi Hospital and Nippon Medical School Hospital between February 2019 and January 2023 and provided written informed consent. However, this study did not include minors. Heart failure, renal failure, pulmonary failure, and malignant diseases were excluded.

Clinical symptoms

The clinical symptoms of patients with FD were estimated based on the Rome Ⅳ criteria [10]. Patients with FD had at least one of the following clinical symptoms: epigastric pain, epigastric burning, postprandial fullness, or early satiety. Clinical symptoms were also assessed using the Gastrointestinal Symptom Rating Scale (GSRS) [11]. Permission to use GSRS was obtained from AstraZeneca by the authors. Information on body mass index (BMI), alcohol consumption, and smoking status was collected using questionnaires (Appendix 1).

Definition of abnormalities of the pancreatic enzymes

Serum levels of pancreatic amylase (p-amylase), lipase, trypsin, phospholipase A2 (PLA2), and elastase-1 were measured using an automated chemistry analyzer (AU5822 analyzer; Beckman Coulter, Brea, CA, USA). The normal ranges of pancreatic enzymes at our hospital were as follows: 18-53 (U/L) for p-amylase, 11-53 (U/L) for lipase, 100-550 (ng/mL) for trypsin, 130-400 (ng/dL) for PLA2, and 0-300 (ng/dL) for elastase-1.

EUS procedure

EUS was performed using Olympus EUS (GF-UCT 260; Olympus, Tokyo, Japan) under sedation in patients with R-FD. Endosonographic features were determined based on a previous study [12]. The EUS score (from zero to four) is estimated by the sum of the EUS findings, such as hyperechoic foci or strands, lobularity, hyperechoic main pancreatic duct (MPD) margin, and dilated side branches. The EUS score was evaluated according to at least two out of four EUS images [12]. More than two expert endoscopists estimated EUS scores, and they were blinded to the clinical status of the patients and genotypes. When there were any differences in opinions among expert endoscopists, the final diagnosis was determined by consensus following a discussion of each case on-site during examination.

DNA extraction and SNP genotyping

DNA was derived from the blood or duodenal samples of the duodenum using a commercial system. Polymerase chain reaction (PCR) was performed on the 7500 Fast PCR System. TaqMan genotyping reactions were carried out in a total volume of 10 μL containing 1× TaqMan™ Genotyping Master Mix, 1× primer (rs1606365 (Assay ID: c_8909099_30) of *GPRC6A*, rs17107315 (Assay ID: c_11157434_10) of *SPINK1*, were ready-made primers, Thermo Fisher Scientific, MA, USA), and 10-ng of DNA. Artificially synthesized positive controls were used for each analysis.

Evaluation of exocrine and endocrine pancreatic function

An N-benzoyl-l-tyrosyl-p-aminobenzoic acid (BT-PABA) test was performed to evaluate the exocrine pancreatic function. Briefly, urine was collected early in the morning after overnight fasting and used as a control. The patients ingested 500 mg BT-PABA (Eisai Co., Ltd., Tokyo, Japan) in 200 mL of water. Exocrine pancreatic failure was defined using a cutoff value of 70%.

Homeostasis model assessment of β-cell function (HOMA-β) was calculated as follows: the sample size was determined using G* Power (version 3.1.9.7, Heinrich-Heine-Universität Düsseldorf, Düsseldorf, Germany). In the analysis of variance (ANOVA) in R-FD, with α = 0.05, 1-β = 0.80, and number of groups = 3, the total number required was 66, and the actual power was 0.818.

Statistical analysis

Two-tailed unpairedt-tests were used to compare the continuous variables between the R-FD and FD groups. Chi-square tests were used to compare categorical variables between the R-FD and FD groups, and between the three groups according to *GPRC6A* genotype. One-way ANOVA was followed by a post-hoc test with Bonferroni correction for continuous variables of the three groups according to *GPRC6A* genotype. All analyses were performed using IBM SPSS Statistics for Windows, Version 27 (Released 2019; IBM Corp., Armonk, New York, United States). Results are presented as mean ± standard error, and p-values < 0.05 were considered statistically significant.

## Results

Comparison of clinical characteristics between patients with R-FD and FD

Table 1 shows the clinical characteristics of the patients with R-FD and FD, with no differences between the two groups with regard to age, gender, or alcohol consumption. The BMI was significantly lower in the R-FD group than in the FD group (p = 0.031). Patients with FD had a significantly higher smoking status (Brinkman index, p = 0.015) compared to those with R-FD.

**Table 1:** Comparison of clinical characteristics between patients with refractory FD and FD Data are means ± SE. Chi-square tests were used to compare gender, and two-tailed unpaired t-tests were used to compare the other continuous variables. P-value: refractory FD vs. FD. BMI in refractory FD was significantly (p < 0.05) lower than that in FD. Smoking in FD was significantly (p < 0.05) higher than that in refractory FD. FD: functional dyspepsia; BMI: body mass index; BI: Brinkman index

Characteristics	Refractory FD	FD	P-value
Age	54.8 ± 2.2	53.8 ± 1.6	0.722
Gender (M/F)	24/50	26/71	0.423
BMI (kg/m^2^)	20.6 ± 0.4	21.8 ± 0.4	0.031
Smoking (BI)	95.9 ± 28.6	276.5 ± 83.6	0.015
Alcohol consumption (g/day)	3.99 ± 2.54	8.67 ± 4.92	0.404

Comparison of pancreatic enzyme abnormalities between patients with R-FD and FD

Pancreatic enzyme levels in the R-FD and FD groups and the percentage of patients with abnormal pancreatic enzyme levels are shown in Table 2. Significant differences (p < 0.001) were observed in the ratio of abnormal pancreatic enzyme levels between patients with R-FD and those with FD (Table 2). Interestingly, trypsin levels may be highly reflected in R-FD than in other pancreatic enzymes. Therefore, measurement of trypsin may be a useful marker for the detection of R-FD.

**Table 2:** Comparison of pancreatic enzyme abnormalities between patients with refractory FD and FD Percentages represent the proportion of patients with pancreatic enzyme abnormalities. Data are means ± SE. Two-tailed unpaired t-tests were used to compare. P-value: refractory FD vs. FD. The ratio of each pancreatic enzyme abnormality in refractory FD was significantly (p < 0.001) higher compared to those in FD. FD: functional dyspepsia, PLA2: phospholipase A2

Enzymes	Refractory FD (%)	FD (%)	P-value
P-amylase (U/L)	52.9 ± 2.2 (43.2)	31.9 ± 0.7 (0.0)	< 0.001
Lipase (U/L)	55.3 ± 3.6 (33.8)	31.3 ± 0.8 (0.0)	< 0.001
Trypsin (ng/mL)	782 ± 109 (83.8)	393 ± 9 (0.0)	< 0.001
PLA2 (ng/dL)	417 ± 14 (54.1)	278 ± 6 (0.0)	< 0.001
Elastase-1 (ng/dL)	156 ± 9 (6.5)	112 ± 5 (0.0)	< 0.001

Distribution of each genotype among the R-FD, FD, and control groups

The distribution of *GPRC6A* and *SPINK1* genotypes in the control group was 43CC (37.1%), 55CG (47.4%), and 18GG (15.5%) and 116AA (100%), respectively (Table 3). The distribution of *GPRC6A* and *SPINK1* genotypes in the R-FD group was as follows: 35CC (47.3%), 31CG (41.9%), and 8GG (10.8%) and 74AA (100%), respectively. The distribution of *GPRC6A* and *SPINK1* genotypes in the FD group was 38CC (39.2%), 45CG (46.4%), and 14GG (14.4%) and 97AA (100%), respectively. No significant differences in the distribution of *GPRC6A* and *SPINK1* genotypes were observed among the three groups (Table 3).

**Table 3:** Distribution of each genotype among refractory FD, FD, and control Chi-square tests were used to compare. P-value: comparison between the three groups. FD: functional dyspepsia

Genotype	Refractory FD	FD	Control	P-value
All (n)	74	97	116	N/A
*GPRC6A *CC (n)	35	38	43	0.683
CG (n)	31	45	55	
GG (n)	8	14	18	
*SPINK1 *AA (n)	74	97	116	N/A

Comparison of blood lipids and glucose tolerances between patients with R-FD and FD

Blood triglyceride levels were significantly higher in the R-FD group than in the FD group (p = 0.045); however, no differences were observed in total cholesterol, low-density lipoprotein (LDL) cholesterol, and high-density lipoprotein (HDL) cholesterol levels (Table 4). No significant difference was observed in glucose tolerance between the two groups.

**Table 4:** Comparison of blood lipids and glucose tolerances between patients with refractory FD and FD Data are means ± SE. Two-tailed unpaired t-tests were used to compare. P-value: refractory FD vs. FD. Blood TG levels of refractory FD were significantly (p <0.05) higher than those of FD. FD: functional dyspepsia; T-Cho: total cholesterol; LDL-Cho: low density lipoprotein cholesterol; HDL-Cho: high density lipoprotein cholesterol; TG: triglyceride; BS: blood sugar; HbA1c: hemoglobin A1c

Parameters	Refractory FD	FD	P-value
T-Cho (mg/dL)	206 ± 4	207 ± 3	0.934
LDL-Cho (mg/dL)	119 ± 4	124 ± 3	0.331
HDL-Cho (mg/dL)	65.9 ± 1.9	65.6 ± 1.9	0.913
TG (mg/dL)	121 ± 8	102 ± 6	0.045
BS (mg/dL)	94.8 ± 1.9	97.7 ± 2.7	0.389
HbA1c (%)	5.64 ± 0.04	5.70 ± 0.07	0.419
Insulin (μU/mL)	11.97 ± 1.38	9.37 ± 1.08	0.140

Comparison of *GPRC6A* genotype with EUS findings in R-FD

Since the *GPRC6A* genotype was reported to be linked to pancreatitis, we tried to investigate the correlation between *GPRC6A* and EUS features in R-FD. EUS score in the GG genotype was significantly higher than that in CC or CG genotypes (*p* = 0.036 and *p* = 0.031, respectively) of *GPRC6A* in R-FD (Table 5). In addition, the lobularity in the GG genotype of *GPRC6A* in R-FD was significantly higher (*p* = 0.023 and *p* = 0.027, respectively) than that in CC or CG (Table 5).

**Table 5:** Comparison of GPRC6A genotype with EUS findings in refractory FD One-way analysis of variance (ANOVA) was followed by a post-hoc test with Bonferroni correction for continuous variables of the three groups. Values are presented as mean ± standard error; values in ( ) are 95% confidence intervals of the mean. p1 value: p-value of EUS total score, p2 value: p-value of lobularity, p3 value: p-value of stranding or hyperechoic foci, p4 value: p-value of hyperechoic MPD, p5 value: p-value of dilated side branches, p-value of CC vs. CG is shown in the top row, p-value of CG vs. GG is shown in the second row, p-value of GG vs. CC is shown in the bottom row. FD: functional dyspepsia; *GPRC6A: G-protein-coupled receptor class C group 6 member A*; EUS: endosonography; MPD: main pancreatic duct

Genotype	EUS total score	Lobularity	Stranding or hyperechoic foci	Hyperechoic MPD	Dilated side branches	p1-value	p2-value	p3-value	p4-value	p5-value
*GPRC6A *CC	0.480 ± 0.102 (0.270 – 0.690)	0.000 ± 0.000 (0.000 – 0.000)	0.200 ± 0.082 (0.030 – 0.370)	0.280 ± 0.092 (0.090 – 0.470)	0.000 ± 0.000 (0.000 – 0.000)	1.000	1.000	1.000	0.960	1.000
CG	0.450 ± 0.135 (0.170 - 0.730)	0.000 ± 0.000 (0.000 – 0.000)	0.250 ± 0.099 (0.040 – 0.460)	0.150 ± 0.082 (-0.020 – 0.320)	0.050 ± 0.050 (-0.050 – 0.150)	0.031	0.027	0.681	1.000	0.599
GG	1.170 ± 0.307 (0.380 – 1.960)	0.170 ± 0.167 (-0.260 – 0.600)	0.500 ± 0.224 (-0.070 – 1.070)	0.330 ± 0.211 (-0.210 – 0.880)	0.170 ± 0.167 (-0.260 – 0.600)	0.036	0.023	0.418	1.000	0.190

Stranding or hyperechoic foci, hyperechoic MPD, and dilated side branches in GG were also higher than those in CC or CG, although the differences were not significant (Table 5).

## Discussion

The major findings of this study were as follows: significant differences were observed (p < 0.001) in the ratio of abnormal pancreatic enzymes between patients with R-FD and FD, and the EUS score in the GG genotype was significantly higher than that in the CC or CG genotypes of *GPRC6A* in R-FD. In addition, the lobularity in the GG genotype of *GPRC6A* in R-FD was significantly higher than that in CC or CG.

R-FD was defined as the lack of improvement in clinical symptoms after two kinds of treatment, such as proton pump inhibitors, vonoprazan, prokinetics, and *H. pylori* eradication. Since significant differences were observed in the ratio of abnormal pancreatic enzymes between patients with R-FD and those with FD (Table 3), we compared the distribution of pancreatitis-related genotypes, such as *GPRC6A* and *SPINK1*. However, no significant difference was observed in the distribution of *GPRC6A* and *SPINK1* genotypes between the R-FD and FD groups. Although there have been many studies on SNPs about FD-related proteins such as G protein subunit beta 3 (GNB3), solute carrier family 6 member 4 (SLC6A4), and cholecystokinin-1 receptor (CCK-1R) [13-17], no previous studies have investigated SNPs related to R-FD. To our knowledge, this is the first study to report R-FD and pancreatitis-related SNPs. In our data, although the *GPRC6A* genotype was not significantly associated with pancreatic enzyme abnormalities of R-FD, the *GPRC6A* genotype was significantly linked to EUS features.

We previously reported that vonoprazan- and acotiamide-resistant FD, referred to as R-FD, exhibited pancreatic enzyme abnormalities [5]. Therefore, we attempted to clarify whether pancreatitis-related genotypes are associated with R-FD. *GPRC6A* and *SPINK1* genotypes were not significantly associated with FD symptoms and pancreatic enzyme abnormalities (Appendices 2, 3). In addition, the *GPRC6A* genotype was not associated with the BT-PABA test and HOMA-β, as exocrine and endocrine pancreatic dysfunction (Appendix 4). We also investigated the relationship between clinical symptoms and *GPRC6A* genotype. Indigestion alone was significantly linked to *GPRC6A* genotypes (Appendix 2). Patients with R-FD tended to have low indigestion scores for the CG genotype. In this study, *GPRC6A* GG genotypes were significantly associated with EUS total score and lobularity, as estimated by EUS. *GPRC6A* is expressed in monocytes and is associated with inflammation [18]. Primary cells isolated from *GPRC6A*-deficient mice exhibit a reduction in calcium-induced secretion of interleukin-1β (IL-1β) [18]. In this study, the *GPRC6A* genotype may lead to EUS features via the elevation of inflammation through the upregulation of IL-1β production. Further studies are required to clarify how *GPRC6A* is associated with EUS features.

This study had several limitations. First, the sample size is relatively small. Since the number of *GPRC6A* GG genotypes was small, further studies will be needed to investigate it precisely in more subjects. However, the prevalence of the *GPRC6A* genotype in East Asia (CC: 44%, CG 42%, GG 14%, *GPRC6A*, National Center for Biotechnology Information (NCBI) database) showed a similar tendency to the *GPRC6A* genotype (CC: 47%, CG 42%, GG 11%) in R-FD in this study. Secondly, we did not investigate the functional consequences of the identified SNPs *GPRC6A* and *SPINK1*. Further studies are required to determine the association between SNPs of *GPRC6A* and *SPINK1* and their activity. Thirdly, in this study, the *GPRC6A* genotype was not significantly associated with clinical symptoms and clinical characteristics of patients with R-FD. Fourthly, we could not identify populations carrying the variant allele of the *SPINK1* gene. Although the prevalence of the variant allele of the *SPINK1* gene was very rare in East Asian populations (A allele was 99.4% and the G allele was 0.6% in rs17107315), further studies will be needed to identify the variant allele of the *SPINK1* gene using more number of populations. Finally, this study was conducted in a Japanese single-center population, and the findings may not be generalizable to other populations.

## Conclusions

There were significant differences in the ratio of abnormal pancreatic enzyme levels between patients with R-FD and those with FD. Therefore, pancreatic enzyme abnormalities may play important roles in the pathophysiology of refractory FD. However, there were no significant differences in the distribution of *GPRC6A* genotypes between refractory FD and FD. Thus, we tried to clarify if EUS features were associated with *GPRC6A* genotypes. The EUS total score and lobularity in the GG genotype were significantly higher than those in the CC or CG genotypes in *GPRC6A* in R-FD.

## Figures and Tables

**Figure 2:**
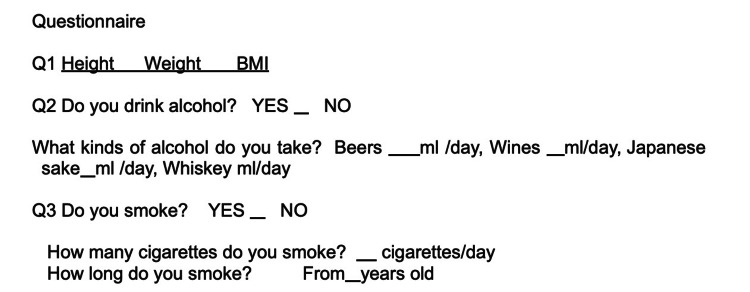
Questionnaire

**Figure 3:**
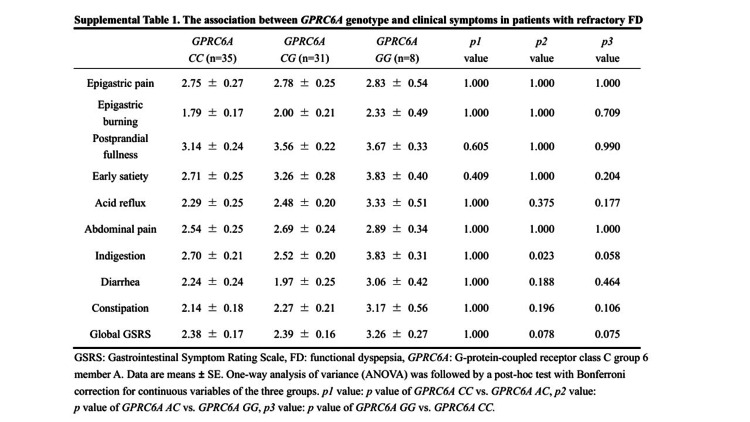
The association between GPRC6A genotype and clinical symptoms in patients with refractory FD

**Figure 4:**
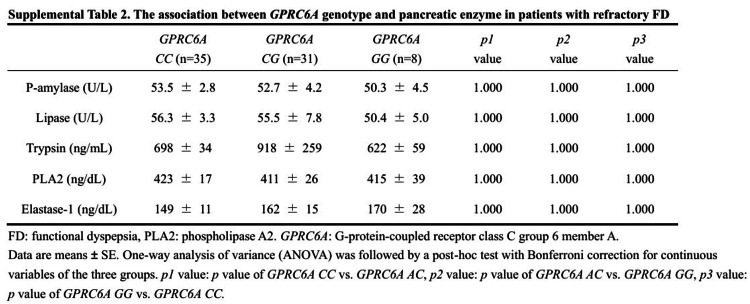
The association between GPRC6A genotype and pancreatic enzymes in patients with refractory FD

**Figure 5:**
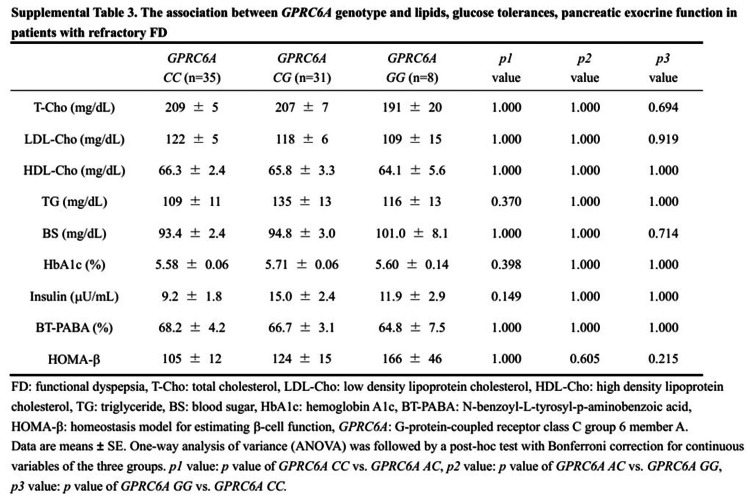
The association between GPRC6A genotype and lipids, glucose tolerances, and pancreatic exocrine functions in patients with refractory FD

## Appendices

Appendix 1

Appendix 2

Appendix 3 

Appendix 4
